# The Potential of Honeybee Products for Biomaterial Applications

**DOI:** 10.3390/biomimetics6010006

**Published:** 2021-01-15

**Authors:** Martina Rossi, Pasquale Marrazzo

**Affiliations:** 1 Department of Pharmacy and Biotechnology, University of Bologna, 40127 Bologna, Italy; martina.rossi12@unibo.it; 2 Department of Experimental, Diagnostic and Specialty Medicine, University of Bologna, 40126 Bologna, Italy

**Keywords:** tissue engineering, honey, biomaterials, antimicrobials, bio-inspired material, propolis

## Abstract

The development of biomaterials required continuous improvements in their properties for new tissue engineering applications. Implants based on biocompatible materials and biomaterial-based dressings are susceptible to infection threat; moreover, target tissues can suffer injuring inflammation. The inclusion of nature-derived bioactive compounds usually offers a suitable strategy to expand or increase the functional properties of biomaterial scaffolds and can even promote tissue healing. Honey is traditionally known for its healing property and is a mixture of phytochemicals that have a proven reputation as antimicrobial, anti-inflammatory, and antioxidant agents. This review discusses on the potential of honey and other honeybee products for biomaterial improvements. Our study illustrates the available and most recent literature reporting the use of these natural products combined with different polymeric scaffolds, to provide original insights in wound healing and other tissue regenerative approaches.

## 1. From the Hive to the Bench

### 1.1. The Beehive Product Profile

The story of honey and other bee products has a very old origin. Honey has been used for thousands of years as a healing treatment around the world. Historic studies revealed that the ancient Egyptians, Greeks, and Romans treated wounds with honey [1]. In recent years, bee products, such as honey, bee bread, bee venom, bee pollen, propolis, and royal jelly, have found new applications both in traditional and modern medicine.

Honey is a by-product of flower nectar and the upper aero-digestive tract of the bee, and is a mixture of a complex chemical composition that varies on botanical source basis [2]. Honey is concentrated through a dehydration process inside the beehive and it is mainly a supersaturated sugar solution. It is composed of 82.4% carbohydrates, 38.5% fructose, 31% glucose, 12.9% other sugars, 17.1% water, 0.5% protein, organic acids, minerals, amino acids, vitamins, phenols, and other minor compounds [3]. The bioactive constituents in honey are phenolic acids, flavonoids, carotenoids, ascorbic acid, α-tocopherol, and proteins, including enzymes (e.g., glucose oxidase and catalase) [3]. Tannins and pigments are also included. A particular, very noteworthy honey is Manuka Honey (MH). MH is produced by honeybees collecting nectar from the *Leptospermum scoparium* shrub, which is indigenous to New Zealand [4,5,6]. The second most principal component produced by honeybees is propolis, which is also generally known as “bee glue.” It is a resinous substance [7]. Its function is that of sealing cracks and holes for the reconstruction of the beehive. It is composed mainly of resin (50%), wax (30%), essential oils (10%), pollen (5%), and other organic compounds (5%), in particular phenolic compounds, flavonoids, terpenes, ascorbic acid, α-tocopherol, beta-steroids, aromatic aldehydes, alcohols, several B vitamins, and minerals [8,9,10]. A few enzymes are also present in propolis [8]. In addition, royal jelly, a white and viscous jelly-like substance, is a form of hypopharyngeal and mandibular gland secretion from worker bees. It is also known as a “superfood” that is solely consumed by the queen bee. It is mainly composed of water (50–60%), proteins (18%), carbohydrates (15%), lipids (3–6%), mineral salts (1.5%), and vitamins [11,12]. In royal jelly, many organic compounds, such as polyphenols, have been detected. Additionally, royal jelly has interesting compounds, such as 10-hydroxy-2-decenoic acid (HAD), which has immunomodulatory properties, royalactin, which is an important functional protein, and hormones such as testosterone, progesterone, prolactin, and estradiol.

### 1.2. The Properties of Honeybee Products: An Ancient Source of Repair

Honey lost popularity after the advent of antibiotics, but it is now experiencing a popular resurgence as a multifactorial antibacterial agent [13], after the venue of antibiotic resistance strains in human pathogenesis. Wound healing application has become the primary field of investigation focusing on this natural product [14]. Intriguingly, honey is produced from the nectar of flowers, which is collected by bees and mixed with insect-secreted enzymes. In fact, honey contains both antioxidant and oxidant molecules. In contrast to the larger potential and in relation to the cost of plant-based antioxidant substances, other natural products and biomaterials can be produced starting from sources external to both the plant and mammalian world. Some of them can complement the human and mammalian native or synthetic extracellular matrix (ECM) panel choice. In this context, “ancient healers,” such as honey, are becoming an attractive natural material for developing biomaterials and may revolutionize tissue engineering [15]. Following this tendency, a new role in regenerative medicine [16] is emerging for honey as biomaterial implementation.

The water activity and the osmotic gradient that can be generated by honey is thanks to its high sugar content; the same typical chemical elements are also involved in desirable cellular adhesion points, which then boost proliferation. The glucose in honey is metabolized by glucose oxidase into hydrogen peroxide and gluconic acid; then, honey modifies the pH, which can contribute to its antimicrobial and immunomodulatory effects.

The wide popularity regarding the healing effect of honey and beehive products most likely derives from its antimicrobial properties. In particular, it is known that the antibacterial activity derives from water deprivation, lowering of pH, reactive oxygen species (ROS), and immunostimulatory molecules; modifying these variables, by changing the floral source, can make a difference in the antimicrobial impact. Firstly, the pH of honey is below 4.5, and thus, is low enough to inhibit the growth of many microorganisms [17] and to increase oxygen release [18]. Secondly, having high osmotic properties, honey can remove water from bacterial cells, and consequently, can prevent the growth of bacteria [18] or kill them [19]. The microbial susceptibility and the experimental inhibition rate can change between Gram-positive and Gram-negative bacteria, because of the presence or absence of an outer membrane that can offer protection against antimicrobial agents, thus making it difficult to penetrate and then damage them [20]. The antimicrobial peptides (AMP) are part of the innate immune system of invertebrates; they are important for the defense against pathogens [21]. The role of Defensin-1, an antimicrobial peptide that can be secreted by honeybee glands, seems to have a synergistic antibacterial effect [22] with other antimicrobial molecules (H_2_O_2_), but is not able to counteract some bacteria alone, for example *Staphylococcus aureus*. Honeybee-derived Defensin-1, able to stimulate secretion of metalloproteinase 9 (MMP-9) from keratinocytes, is effective in wound re-epithelialization in vitro and in vivo [23]. The Major Royal Jelly Proteins (MRJPs) play an essential nutritional role in the diet of the queen bee, some of them are major allergens in royal jelly [22]. MRJP-1 also known as apalbumin-1 is one member of this family. The glycoproteins with high-mannose structure contributed to honey antibacterial effects as well as MRJP-1 precursor in royal jelly; indeed, they share peptide homology. The jelleines [24] that are in royal jelly demonstrated an antibacterial effect in vitro [25]. Jellein 1 and 2 are particularly effective as antimicrobials, while the Royalisin, a defensin-like protein, is active against Gram-positive bacteria [26].

Specifically, Manuka Honey (MH) has been shown to exhibit additional properties against infection, which are attributed to the presence of methylglyoxal (MGO), a glycation inducer. MGO is derived by nonenzymatic conversion of dihydroxyacetone, which is present at high levels in the *L. scoparium* flower’s nectar. Together with the antiseptic effect of hydrogen peroxide, MGO increased the antibacterial effect in MH [27] expressed by the Unique Manuka Factor (UMF). A lot of wound care products are based on MH and are commercially available [1], for example clinical alginate film containing MH, or paste and dressings for topical application. The geometry factor and scaffold formulation can influence the setup of a specific release of MH [28]. In healthy individuals, UMF 20+ has been evaluated as safe [29]. However, it should be not excluded that other honey varieties may be preferable in respect to MH for wide regenerative applications, avoiding any cytotoxicity in mucosae and specific tissues such as neural and muscle ones.

In parallel, the ability to modulate the activity of inflammatory cells could increase the use of honey as a scaffold additive for wound healing and bone infection [30]. Honey possesses stimulatory activity targeting pro-inflammatory cytokines, thus moving wound healing past an extended chronic inflammatory phase [31]. The anti-inflammatory activities by honey flavonoids and proteins were also reported. Honey inhibited the release of Tumor Necrosis Factor-alpha (TNF-α) and Interleukin 1-beta (IL1-β) by microglia cell line stimulated with lipolysaccharide (LPS) [32], the production of TNF-α by human monocytic cells, and the release of ROS from murine macrophages and human neutrophils activated with zymosan [33]. The propolis restored the proliferation capacity and chemotaxis of B and T lymphocytes in diabetic mice model [34]. In propolis, caffeic acid phenethyl ester (CAPE) is a powerful agent that influences the relative antimicrobial and anti-inflammatory activity. Furthermore, MRJP-1 can inhibit phagocytic cells activity by blocking the mannose receptor while MRJP-3 suppressed IL-2, IL-4, and IFN-γ expression by antigen-stimulated T cells [35] and induced TNF-α and MMP-9 levels in keratinocytes [36]. The carboxylic acids from royal jelly, identified as being capable of antimicrobial activity, can reduce Nuclear Factor kappa-B (NF-κB) activation induced by LPS in the macrophage cell line [37].

An abnormal levels of radicals can trigger long-lasting inflammation; indeed, oxidative stress and inflammation are closely related [38]. With regards to oxidative stress protection, the phenolic content of honey can quench oxidative harmful action [39]. Because of the complex nature of similar animal-derived products, after the introduction in a biomaterial it is, unfortunately, hard to distinguish single or combined antioxidant activity realized by specific honey components. Additionally, the propolis has reparative functions [34] and antioxidant activity; the latter is possible thanks to the occurrence of flavonoid and phenolic compounds [40]. Different propolis extract volumes were used to fabricate silver nanoparticles, which retained antioxidant activity and interfered with bacterial biofilm formation according to increasing volumes of the natural compound [41]. In future research, it will be significant to intensify the effort in an almost neglected antiviral capacity [42] of honeybee products in parallel with the most known antibacterial one [13].

## 2. Scaffold Design and Experimental Studies

Instead of a customary fibrotic healing, an optimal repair could be obtained if dermal reconstruction, proper re-epithelialization, and the formation of granulation tissue will occur. To achieve these mandatory steps, researchers can choose between different types of scaffolds, by selecting suitable geometries and physico-chemical properties. By changing proper parameters during the fabrication, the chosen type will be appropriate for enabling the scaffold to be applied in specific or wide tissue engineering strategies. Gels or fibers made by electrospinning are the most studied and are the currently available options for the construction of beehive product-infused or -impregnated biomaterials. While hydrogel- and cryogel-based scaffolds have been used more for muscle, bone, and cartilage tissue applications, electrospun scaffolds are more often applied to skin-related healing [28]. Silk fibroin (SF) is a structural fibrous protein obtained from the cocoon of *Bombyx mori*. The increasing use of SF for electrospinning [43] is promising for the design of porous scaffold and nanofibers [44] for bone [45] and cartilage [46] tissue engineering. Derived from deacetylated chitin, chitosan (CH) has been frequently employed because of its intrinsic antibacterial properties and has been shown to enhance the production of the extracellular matrix, owing to high hydrophilicity. Moreover, CH degradation products (e.g., *N*-acetyl-d-glucosamine) promote fibroblast growth.

The direct addition of pure honey (or other beehive products) may be toxic to cells and tissues, but the use of a substantial vehicle, such as the mentioned scaffolds, can be effective to deliver a controlled, safe and bioactive release of honey bioactive compounds. Part of the investigation is often directed to find the optimal concentration to incorporate such bioactive compounds into a specific scaffold, depending on the used polymer and with the aim to realize the most beneficial effects (i.e., cell infiltration and proliferation, modulation of inflammation and ROS, infection prevention, and tissue regeneration), while maintaining ideal biomechanical properties. For example, as honey is a sugar solution that includes both hydrophilic and hydrophobic properties, the charge of the raw material can influence the hydrophilic part modulating the swelling behavior of the final honey-including scaffold. The swelling ability of honey scaffolds will, however, depend on a sum of factors, including the cross-linking agent and the nature of the materials used and mixed together with honey components. A controlled release over time represents a characteristic that encourages the development of honey-incorporated grafts for clinical use. Indeed, slow and prolonged concentrations of honey will be requested in situ for grafts in organs with an environmental risk exposure (bacterial pathogen and oxygen levels) different from the skin one. Lastly, the opportunity to associate honeybee products with a 3D physiological environment for cell culture, such as into a cellular scaffold, may also be useful to model human in vitro infections [47], which enables more relevant tests of their preventive and therapeutic effects.

Table 1 summarizes the studies reporting the combined use of honeybee products and different types of fabricated scaffolds; the same studies are detailed below in this review.

### 2.1. Gel Scaffolds

Hydrogels are crosslinked polymer networks that store large amounts of water [73] and result in nanoporous gel-like structures [14]. On the basis of the cross-linking methods, polymeric gels can be classified into: physical gels and covalently cross-linked gels [74]. The cryogels are produced similarly to the hydrogel, but the cross-linked polymer solution is quickly frozen and the gelation happens at subzero temperature [74]. During the fabrication, the ice crystals formed throughout and after thawing melt out of the scaffold, thus generating a macroporous spongy-like structure [31]. The result is a highly porous scaffold [30], which allows cell migration inside it, usually more easily than into a hydrogel. Natural polysaccharide-based matrices, such as gums, alginate, and chitosan, exhibit a low immunogenic profile [75] and can be easily manipulated.

In 2016 [49], in search of novel antimicrobial strategies for wound healing, a study focused on modeling and optimizing the antibacterial activity provided by honey and gelatin included in hydrogel films composed of chitosan and poly(vinyl alcohol) (PVA). Thyme honey has been added in range of 0–0.04 g/mL. It showed a significant effect on the antibacterial properties of the hydrogel films against both *Pseudomonas aeruginosa* and *S. aureus*, investigated by agar inhibition zone. For both bacteria, the antibacterial effect of chitosan, PVA, and honey changed according to their concentration, while gelatin concentration did not show an antibacterial effect. The swelling property, analyzed during optimization and measured in films with constant amount of PVA and gelatin, could be related to changes of chitosan or honey. An experimental increase in honey concentration led to a major mechanical strength as well as reduction in water absorption, while chitosan was the main controller of the swelling extent.

In order to enhance mechanical properties and prevent infections, fabrication of scaffold based on gellan gum and MH was explored to support cartilage healing. Human Mesenchymal Stem Cells (hMSCs) can adhere to the scaffold surface and, after 21 days of culture, differentiate in chondroblast; in addition, the biomaterial did not show cytotoxicity even without direct cell contacts. The antibacterial activity of the hydrogels in terms of reduced biofilm *S. aureus* and *Staphylococcus epidermidis* viability was proven. The release of MGO could be modulated by calcium or magnesium cross-linking of the same scaffolds [75].

Aiming to the optimization of a scaffold improved for cartilage tissue engineering, the authors of the same study described the preparation of composite scaffolds, based on gellam gum and MH, including three different inorganic clays [62]. The mesoporous silica scaffold offered, together with MH, an antibacterial effect and protected hMSCs co-cultured with staphylococcal strains. The clay did not alter the degradation rate of the MH-loaded scaffold, which allowed growth and chondrogenic differentiation of the stem cells in vitro. When these scaffolds were introduced subcutaneously in mice, they did not cause severe immunological reaction and were effective in enhancing the antibacterial response.

MH (2% *w*/*v*) can be used for the preparation of a porous composite scaffold based on gellan gum and resveratrol, a polyphenolic compound, loaded in diatom silica shells [63]. In vitro tests confirmed the antioxidant activity and the antibacterial properties of the composite scaffolds that demonstrated mechanical properties suitable for cartilage regeneration.

Manuka Honey (MH) silk fibroin cryogels were analyzed in comparison to MH gelatin cryogels [30]. The incorporation of MH (at 1%, 5%, and 10%) had a significant negative impact on gelatin cryogels, at the level of the swelling potential, and caused a decrease of mechanical properties. The addition of MH within the single material maintained the macroporous structure suitable for cell compatibility. The MH silk fibroin scaffolds had highest porosity and released more MH than gelatin scaffolds. In summary, 5% MH cryogel promoted bacterial clearance (also in liquid cultures) of *E. coli, S. agalactiae* (or group *B. streptococcus*), and *S. aureus*, which are bacteria shown to cause chronic bone infection, thanks to a sustained release of antimicrobial products. To evaluate the osteoconduction properties that are advantageous in bone defect applications, the cellular proliferation, infiltration, and cryogel mineralization by osteosarcoma derived-cell line was studied. Overall, the MH did not impair mineralization, and silk fibroin was superior for cell proliferation.

In 2017, a composite hydrogel sheet was designed to make a dressing, showing desirable properties for wound healing, with chitosan as the primary functional material, suggesting to promote ordered dermal regeneration. For enhancing antibacterial and healing properties chitosan was combined with sunflower honey (20 wt%), and, additionally, with bovin gelatin owing to its filmogenic activity and absorbing capacity [51]. The sheet capacity of retaining water decreased with increasing honey concentrations that were tested. The data related to the swelling degree in phosphate buffered saline (PBS) agreed with Young’s modulus measurements; indeed, the moduli in samples with honey decreased in comparison to control sheet without honey. *E. coli* and *S. aureus* were investigated as in vitro infection models, and the inhibition rates to bacteria demonstrated a synergistic effect between chitosan and honey. The healing time of rabbit skin burns treated with the hydrogel sheet was faster if compared with a commercially available ointment or untreated controls. The authors of the study also reported good wound contraction and hair follicles proliferation by the hydrogel sheet. However, together with water uptake, there was a limit in the novel application, i.e., the remaining quantity of inflammatory cells. This might be associated with the solvent used for chitosan in the biofabrication.

In a recent study, chicory honey (0–20% *v*/*v*) was added to polymer solution to make a final freeze-dried hydrogel composed of chitosan, PVA, and gelatin (2:1:1) [18]. In this study, the elastic modulus and tensile strength of the gels were reduced by increasing the concentrations of honey—the pore size was increased and the degradation rate was faster than in controls with absence or lower concentrations of honey. The antibacterial activity against *S. aureus* and *P. aeruginosa* was higher in honey-loaded samples than controls. The proliferation of fibroblasts in vitro was supported in a dose-dependent manner until a honey concentration of 10%, in comparison to the pristine scaffold. In a rat wound model, honey-containing hydrogels showed accelerated wound closure, re-epithelialization, transition from inflammation to proliferation during healing, and mature collagen expression.

In another study, a sodium alginate-based hydrogel was coupled with honey (2–10%). For improving the stability of this hydrogel, both ionic and covalent cross-linking were performed. The stiffness of the honey-blended hydrogels decreased with the increase in honey concentration, as well as its surface hydrophilicity, analyzed by contact angle measurements. The swelling index was correlated with increased honey concentration; however, a specific honey embedded concentration was shown to be the best for controlled degradation. The investigators commented that amylase and α-glucosidase from honey might be responsible for weakening covalent cross-linking resulting into the decreased stiffness. The addition of 4% honey provided the best results among all experimental groups in antimicrobial activity against *S. aureus* methicillin-resistant strains and *E. coli*, suggesting the roles played by glucose oxidase and myeloperoxidase, enzymes included in honey. Here, honey-stimulated cell metabolic activity of both keratinocytes and fibroblasts with spindle-like morphology. The in vivo analysis of changes in wound closure kinetics in a rat wound healing model, granulation tissue formation, collagen fibers, and fibroblast density finally suggested that 4% honey concentrations were able to favor a healing environment, because the honey-treated group resembled more closely the normal skin epithelium, matrix, and even hair follicles [50].

Topical hydrogels were prepared using honey and CH or carbopol 934. In particular, the CH honey gel with respect to single honey or blank CH was a better healer at the end of the antimicrobial assay against burn infection bacterial strains and in histopathological analyses of burn wounds in vivo [52].

Carboxymethyl cellulose was successfully used as a chestnut honey carrier to be evaluated as a competitive candidate for the treatment of diabetic ulcer wounds [53]. Water uptake by these hydrogels and their ability to inhibit and kill bacteria was correlated with honey percentages, and a methylglyoxal relation was hypothesized because of its abundance in the used chestnut honey. In vivo acceleration of dermal repair and modulation of timely behavior in granulation tissues was shown to be significantly different in honey samples.

Pectin, a heterosaccharide present in the plant cell wall, has recently been investigated for use in different biomedical applications, such as drug delivery, skin protection, and scaffolding. In this study, pectin-MH hydrogel showed good fluid uptake and were not cytotoxic for fibroblasts. They were found to be effective against bacterial common human pathogens [60], and in vivo, no histological changes were observed after rat subcutaneous implantation. The combination was superior to a pectin-only scaffold or fluid honey-alone application [61].

A biodegradable hydrogel was fabricated by using β-cyclodextrin (β-CD) and κ-carrageenan for delivery encapsulated propolis extract. The great antibacterial (*P. aeruginosa* and *S. aureus*) and antifungal activity provided to the hydrogel increased by increasing the concentration of the encapsulated propolis [67].

The concurrent use of royal jelly with hydroxyapatite for bone healing has been reported; however, relevant literature is lacking in the combined use of royal jelly with biomaterials [76]. Very recently, extracellular vesicles of royal jelly were incorporated in a type I collagen matrix [72]. The released vesicles modulated the capacity of fibroblasts to migrate in vitro and were effective against *S. aureus* biofilm formation, thus demonstrating a potential delivery system for wound healing therapies.

Initially intended for industrial application, a hydrogel of poly(ethylene glycol) diacrylate (PEGDA) was developed introducing a honey-inspired peroxide-producing enzyme: glucose oxidase. Not only does the enzyme have long-lasting activity into the gel network, but the antimicrobial activity against *S. epidermidis* of the loaded hydrogel increased considerably [66].

### 2.2. Electrospun Scaffolds

We noticed that the incorporation into electrospun fibers is the most emerging method for utilizing honey in tissue-engineered scaffolds, at least for wound healing applications. The final nonwoven mesh structure, the flat geometry, the mechanical properties, and the capacity to absorb the exudate (watery consistency) encourage the choice of this scaffold type. Briefly, in the electrospinning technique, the polymer solution is pushed through a syringe after a high-intensity electric field is applied to a conductive needle [31]. When the voltage is applied at the needle tip, the solvent evaporates during the time of flight toward the grounded target, where the polymeric scaffold is collected [57]. Sometimes, the bad side of the coin could be the requirement of a toxic solvent.

Poly(1,4-cyclohexane dimethylene isosorbide treph- thalate) (PICT) is a synthetic and biodegradable polymer [48]. Three blend ratios of PICT/Honey were used 90:10, 85:15, and 80:20 to develop antimicrobial electrospun nanofibers. The second ratio has been suggested to be the most suitable option, because of elastic behavior and tensile strength, in comparison to others. In particular, larger diameters were detected with increasing honey quantity; furthermore, the hydrophobicity was decreased.

MH was used as a cross-linking agent to fabricate fiber mats of only poly(ε-caprolactone) (PCL) or blend fibers of PCL and methylcellulose (MC) by electrospinning [57]. These fibers were considered useful as biodegradable platform to potentially deliver bioactive glass particles (BG). The PCL fibers containing both MH and BG enhanced the release rate of MH, while the corresponding blend fibers showed a lower release rate of MH compared to the fibers without BG. The addition of MH caused an increase in fiber diameter and a reduction of tensile strength for both types of fibers. MH did not significantly change cell viability of dermal fibroblasts in blend fibers, but improved migration of keratinocyte-like cells in scratch assay. No antibacterial effect was demonstrated by such fabricated samples against *E. coli* and *S. aureus* in liquid cultures.

Scaffolds based on SF were evaluated merely in vitro after the incorporation of MH [31]. In general, UMF variation in fixed 5% MH concentration did not cause a difference in the antibacterial effect between its use in cryogel or in electrospun fibers that were based on an identical material [31]. In that case, fibroblast cytotoxicity was only detected when cell medium was supplemented with MH instead of cell media conditioned by honey-loaded scaffolds.

Recently, in a comparative study [28], Hixon et al. mainly focused on the relationship between the geometry of silk fibroin- based scaffolds and the antibacterial effects induced by MH. Electrospun scaffolds in respect to hydrogels and cryogels faster released the MH, thus leading to a superior *S. aureus* clearance and adhesion.

Several human fibroblast and endothelial primary cells were cultured on PCL biomimetic nanofibers coated with MH [20]. Their viability depended on the concentration of honey, and some concentrations increased cell proliferation in comparison to clean PCL mesh. Beware of his double-faced nature; further research will be needed to understand the inflammatory and in vivo response to MH-loaded biomaterial. The antimicrobial effect induced by MH was dependent on its concentration and bacterial strains.

SF nanofibers were fabricated with poloxamer 407 or with MH. When compared to each other, the density of human dermal fibroblasts was shown to be higher in the honey scaffold [58].

Studies about the incorporation of MH (1–20% *v*/*v*) in a PCL nanofiber scaffold showed that it could increase the fibroblast infiltration into the scaffold and proliferation [59] and suggested honey functionalization for wound healing application. Honey incorporation did not increase the degradation rate of the scaffolds, and some of them were effective against *E. coli* growth.

### 2.3. Other Scaffolds

Foams are other scaffolds that can be utilized in tissue engineering. Freeze-dried foams based on methylcellulose were cross-linked with MH [64]. The MH reduced the contact angle, leading to high hydrophilicity and improved wettability. The viability of normal or cell line-derived fibroblasts cultured on top of the scaffold with MH was comparable to controls. The results of a scratch assay showed that the migration ratio of keratinocytes-like cells and antibacterial activity against *E. coli* and *S. aureus* was enhanced for both wound healing-relevant bacteria. For wound healing applications, not only mechanical properties can be improved by the functional addition of borate bioactive glass. Indeed, the release of MH and the killing of bacteria was enhanced after the inclusion of specific bioactive glass; however, further investigation will be needed for finding optimal non-toxic concentrations.

Within its self-assembly, the properties of a collagen scaffold, in particular collagen denaturation and tensile strength, can be reduced by impregnation and following deposition on fibrils of propolis nanoparticles [68]. At 100 μg/mL, the propolis obtained from *Apis mellifera* maintained the fibril structure of collagen extracted from rat tail and enabled the culture of fibroblast cell line, by providing cells a metabolic stimulus, likely activating their proliferation.

A recent study comprising pre-clinical results analyzed polyurethane (PU) foams that improved in vitro L929 fibroblast compatibility and in vivo wound healing, together with showing broad antibacterial properties; all the effects were associated with the water propolis extract [70].

A study characterizing a scaffold of PU incorporating propolis into its fibers showed that the mat increased its hydrophilicity and its fibroblast compatibility and carried out an effective antibacterial activity [65]. A biomaterial based on shell propolis helped human dental stem cells in vitro osteogenic differentiation [69]; future in vivo studies possibly will define it as a suitable material for bioactive cements and for orthopedic material development.

Furthermore, PLA-PCL-propolis composite films were proposed as a possible material for periodontal-guided tissue regeneration [71].

Very recently, using freeze-drying technique, the incorporation of honey in silk fibroin 3D scaffolds was analyzed for the development of a wound healing substrate that can act as a dermal substitute [55]. Honey was added with the hypothesis of enhancing water retention and facilitating cell migration. Stable foam scaffolds were obtained with a honey concentration from 1% to 6% (*v*/*v*). With the increase of honey, the pore size, the swelling ratio, and the degradation rate increased, while pore density decreased. Honey stimulated the proliferation of primary fibroblasts. In vitro, the 4% honey concentration was the best in enhancing the synthesis of ECM proteins by the cells infiltrated within the scaffolds, e.g., producing collagen I, collagen III, α-smooth muscle actin (α-SMA), and fibronectin. When the 4% honey scaffold was implanted subcutaneously in a rat model, it fastened the wound healing process. Minimal scarring, physiological epithelialization, and the presence of hair follicles and blood vessels defined this scaffold as the best candidate for skin repair. Interestingly, hypoxia-inducible factor-1α (Hif-1α) protein expression in such in vivo-treated sample was lower than normal skin control or sham; this suggests that Hif-1α could be involved in the oxygen-dependent collagen synthesis and angiogenesis stimulation.

An MH-PVA-based dressing enabled a sustained release of honey and worked as an antibacterial and fibroblast proliferator. Furthermore, erythromycin loading into the dressing was allowed, and the antibiotic release was not affected by honey [54]. An antibacterial effect was also detected against *S. aureus*.

Bioprinting is a promising technique to fabricate blended scaffolds, and many polymers are printable as bioinks. The loading with honey into printed scaffolds improved 3T3 fibroblast proliferation in the presence of blended alginate scaffold in comparison to pure alginate [56].

## 3. Concluding Remarks

Products of natural origin, such as honey, propolis, and royal jelly, should be taken into consideration for new therapeutic applications in regenerative medicine. One main reason is their availability, based on geographical areas, and, especially in developing countries, its low cost in comparison to synthetic similar products [34]. In the last decade, the number of studies describing biomaterials enriched by the honeybee products that have demonstrated antimicrobial, regenerative, and immunomodulatory effects has gradually increased. The variable quality and quantity of phenolics are significant contributors to a wide range of biological effects and antioxidant capacity in honey of different origins [77]. This antioxidant potential may be useful for developing tunable systems that sense and regulate the oxidative stress level after the implantation of tissue-engineered constructs [78]. However, honey variation is great and depends on many factors, such as the seasonal collection time and botanical source. Currently, hydrogel and electrospun scaffolds are very popular in the field of tissue engineering, but relative few studies have investigated the potential of honey, propolis, or royal jelly as bioactive functionalization agents, perhaps because of the variability mentioned above. Standardization of the levels of the antimicrobial compounds, which are usually antioxidants, is demanded for the confident applicability of honeybee products for bioengineering purposes. Indeed, the use of standardized preparation or isolated components would positively contribute to the level of control of biophysical and biological modifications of the scaffolds, requested by different tissues.

**Figure 1:**
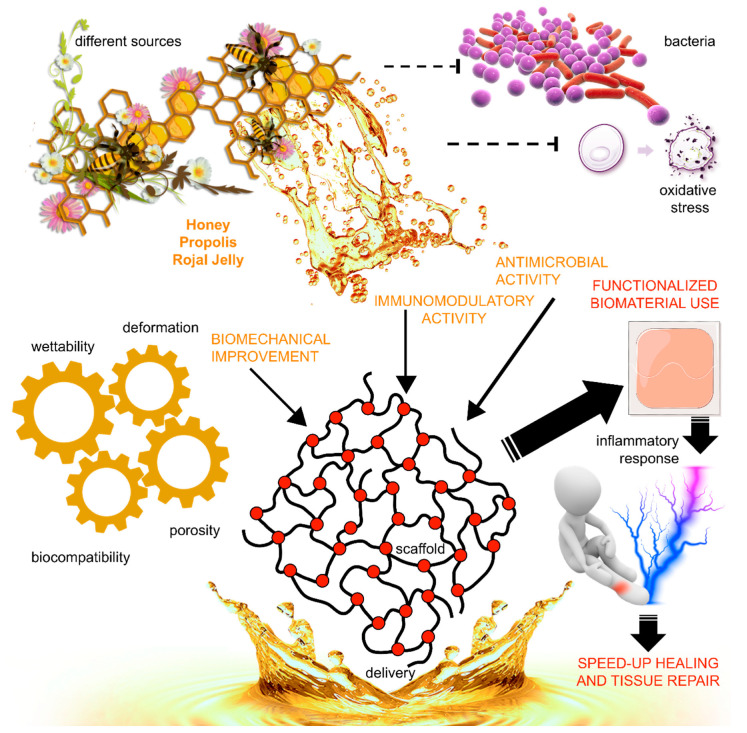
Overview of the advantages of using honeybee products in tissue engineering. Honeybee products, such as honey, propolis, and royal jelly, differ according to the botanical source and the geographical origin. Importantly, they share bioactive compounds with significant antimicrobial, antioxidant, and immunomodulatory effects, suitable for modifying functional properties of biomaterials. These features can be transferred to a scaffold for biomedical application in order to adapt its mechanical properties, to avoid its rapid degradation, bacterial infection, and cell toxicity, and to allow a local delivery. The application or the transplantation in the body of the scaffold functionalized by honeybee products can favor the healing and the repair of injured tissues, by indirectly modulating the inflammatory response.

The pro-healing stimuli delivered by the honey-loaded scaffolds can soon revolutionize the therapeutic vision of honey and other honeybee products (Figure 1). 

Some examples of clinical applications of honey-loaded materials already exist in dermatology. Spongy pads of type I collagen impregnated with medical-grade honey, namely Collagen Revamil^®^, or dressing based on calcium alginate, such as Melginate^®^ or Algivon^®^, exploit the healing potential of honey [1]. In future research, it will be important to intensify the effort in researching the antiviral capacity [42] of honeybee products in parallel with the most known antibacterial one [13].

## Figures and Tables

**Table 1:** Experimental studies on biomaterials functionalized with honeybee products analyzed in this review.

Incorporate Product	Scaffold Type	Building Blocks (e.g., Polymers, Blends, etc.)	Antibacterial Effect	Biocompatibility and Cell Proliferation	In Vivo Healing Effect	Reference
Honey	Electrospun	PICT	NA	NA	NA	Khan et al., 2017 [48]
	Hydrogel	Chitosan, PVA, Gelatin	Yes	NA	NA	Lahooti et al., 2016 [49]
	Hydrogel	Alginate	Yes	Fibroblasts	Yes	Mukhopadhyay et al., 2020 [50]
	Hydrogel	Chitosan, gelatin, PVA	Yes	Fibroblasts	Yes	Shamloo et al., 2020 [18]
	Hydrogel	Chitosan gelatin	Yes	NA	Yes	Wang et al., 2012 [51]
	Hydrogel	Carbopol 934 and Chitosan	Yes	NA	Yes	El-Kased et al., 2017 [52]
	Hydrogel	Carboxymethyl cellulose	Yes	No	Yes	Park et al., 2017 [53]
	Hydrogel	PVA	Yes	Fibroblasts	NA	Tavakoli et al., 2017 [54]
	Scaffold	Silk Fibroin	NA	Fibroblasts	Yes	Rajput et al. 2020 [55]
	Bioink	Alginate	NA	Fibroblasts	NA	Datta et al., 2018 [56]
Manuka Honey	Electrospun	PCL	Yes	Fibroblasts and endothelial cells	NA	Mancuso et al., 2019 [20]
	Electrospun	PCL, MC, BG	Yes	Fibroblasts	NA	Schuhlanden et al., 2020 [57]
	Electrospun	Silk fibroin Poloxamer 407	NA	Fibroblasts	NA	Kadakia et al. 2017 [58]
	Electrospun	PCL	Yes	Fibroblasts	NA	Minden-Birkenmaier et al., 2018 [59]
	Electrospun and Cryogel	Silk fibroin	Yes	Fibroblasts	NA	Hixon et al., 2017 [31]
	Hydrogel, Cryogel and Electrospun	Silk Fibroin	Yes	NA	NA	Hixon et al., 2019 [28]
	Cryogel	Gelatin, Silk Fibroin	Yes	MG-63	NA	Hixon et al., 2018 [30]
	Hydrogel	Pectin	Yes	L292	NA	Giusto et al., 2018 [60]
	Hydrogel	Pectin	NA	NA	Yes	Giusto et al., 2017 [61]
	Hydrogel	Gellam gum	Yes	hMSCs	NA	Bonifacio et al., 2020 [62]
	Hydrogel	Gellam gum DE/RESV	Yes	hMSCs	NA	Bonifacio et al., 2020 [63]
	Foam	bioactive glass methylcellulose	Yes	Fibroblasts	NA	Schuhladen et al., 2020 [64]
Propolis	Electrospun	PU	Yes	Fibroblasts	NA	Kim et al., 2014 [65]
	Hydrogel	PEGDA	Yes	NA	NA	Zhang et al., 2020 [66]
	Hydrogel	(cotton fabric treated with) carrageenan/cyclodextrin	Yes	NA	NA	Sharaf et al., 2019 [67]
	Other	Collagen	NA	Fibroblasts	NA	Gonzales-Masis et al., 2020 [68]
	Other	shell clam	NA	hMSCs	NA	Simu et al., 2018 [69]
	Foam	PU	Yes	L292	Yes	Khodabakhshi et al., 2019 [70]
	Film	PLA and PCL	Yes	NA	NA	Ahi et al., 2019 [71]
Royal Jelly	Hydrogel	Collagen I	Yes	Fibroblasts	NA	Ramirez et al., 2020 [72]

NA (not available), PEGDA (Poly(ethylene glycol) diacrylate), PICT (poly(1,4-cyclohexane dimethylene isosorbide terephthalate), PVA (poly(vinyl alcohol)), PCL (poly(ϵ-caprolactone)), PLA (Poly(L-lactide)), MC (Methylcellulose), BG (Bioactive glasses), PU (polyurethane).

## Data Availability

Not applicable.

## References

[1] Minden-Birkenmaier B.A., Bowlin G.L. (2018). Honey-based templates in wound healing and tissue engineering. Bioengineering.

[2] Eteraf-Oskouei T., Najafi M. (2013). Traditional and modern uses of natural honey in human diseases: A review. Iran. J. Basic Med. Sci..

[3] Pasupuleti V.R., Sammugam L., Ramesh N., Gan S.H. (2017). Honey, propolis, and Royal Jelly: A comprehensive review of their biological actions and health benefits. Oxidative Med. Cell. Longev..

[4] Tomblin V., Ferguson L.R., Han D.Y., Murray P., Schlothauer R. (2014). Potential pathway of anti-inflammatory effect by New Zealand honeys. Int. J. Gen. Med..

[5] Speer S.L., Schreyack G.E., Bowlin G.L. (2015). Manuka honey: A tissue engineering essential ingredient. J. Tissue Sci. Eng..

[6] Jenkins R., Burton N., Cooper R. (2011). Effect of Manuka honey on the expression of universal stress protein A in meticillin-resistant *Staphylococcus aureus*. Int. J. Antimicrob. Agents.

[7] Viuda-Martos M., Ruiz-Navajas Y., Fernández-López J., Pérez-Álvarez J. (2008). Functional properties of honey, propolis, and Royal Jelly. J. Food Sci..

[8] Azzini E., Giacometti J., Russo G.L. (2017). Antioxidant phytochemicals at the pharma-nutrition interface. Oxidative Med. Cell. Longev..

[9] Gómez-Caravaca A.M., Gómez-Romero M., Arráez-Román D., Segura-Carretero A., Fernández-Gutiérrez A. (2006). Advances in the analysis of phenolic compounds in products derived from bees. J. Pharm. Biomed. Anal..

[10] Anjum S.I., Ullah A., Khan K.A., Attaullah M., Khan H., Ali H., Bashir M.A., Tahir M., Ansari M.J., Ghramh H.A. (2019). Composition and functional properties of propolis (bee glue): A review. Saudi J. Biol. Sci..

[11] Nagai T., Inoue R., Suzuki N., Nagashima T. (2006). Antioxidant properties of enzymatic hydrolysates from Royal Jelly. J. Med. Food.

[12] Fratini F., Cilia G., Mancini S., Felicioli A. (2016). Royal Jelly: An ancient remedy with remarkable antibacterial properties. Microbiol. Res..

[13] Kwakman P.H.S., Zaat S.A.J. (2011). Antibacterial components of honey. IUBMB Life.

[14] Hixon K.R., Klein R.C., Eberlin C.T., Linder H.R., Ona W.J., Gonzalez H., Sell S.A. (2019). A critical review and perspective of honey in tissue engineering and clinical wound healing. Adv. Wound Care.

[15] Abbott R.D., Kaplan D.L. (2016). Engineering biomaterials for enhanced tissue regeneration. Curr. Stem Cell Rep..

[16] Martinotti S., Ranzato E. (2018). Honey, wound repair and regenerative medicine. J. Funct. Biomater..

[17] Owayss A.A., Elbanna K., Iqbal J., Abulreesh H.H., Organji S.R., Raweh H.S.A., AlQarni A.S. (2019). In vitro antimicrobial activities of Saudi honeys originating from *Ziziphus spina-christi* L. and *Acacia gerrardii* Benth. trees. Food Sci. Nutr..

[18] Shamloo A., Aghababaie Z., Afjoul H., Jami M., Bidgoli M.R., Vossoughi M., Ramazani A., Kamyabhesari K. (2020). Fabrication and evaluation of chitosan/gelatin/PVA hydrogel incorporating honey for wound healing applications: An in vitro, in vivo study. Int. J. Pharm..

[19] Basa B., Belay W., Tilahun A., Teshale A. (2016). Review on medicinal value of honeybee products: Apitherapy. Adv. Biol. Res..

[20] Mancuso E., Tonda-Turo C., Ceresa C., Pensabene V., Connell S.D., Fracchia L., Gentile P. (2019). Potential of Manuka honey as a natural polyelectrolyte to develop biomimetic nanostructured meshes with antimicrobial properties. Front. Bioeng. Biotechnol..

[21] Marrazzo P., Crupi A.N., Alviano F., Teodori L., Bonsi L. (2019). Exploring the roles of MSCs in infections: Focus on bacterial diseases. J. Mol. Med..

[22] Cornara L., Biagi M., Xiao J., Burlando B. (2017). Therapeutic properties of bioactive compounds from different honeybee products. Front. Pharmacol..

[23] Bucekova M., Sojka M., Valachova I., Martinotti S., Ranzato E., Szep Z., Majtan V., Klaudiny J., Majtan J. (2017). Bee-derived antibacterial peptide, defensin-1, promotes wound re-epithelialisation in vitro and in vivo. Sci. Rep..

[24] Fontana R., Mendes M.A., De Souza B.M., Konno K., César L.M.M., Malaspina O., Palma M.S. (2004). Jelleines: A family of antimicrobial peptides from the Royal Jelly of honeybees (*Apis mellifera*). Peptides.

[25] Romanelli A., Moggio L., Montella R.C., Campiglia P., Iannaccone M., Capuano F., Pedone C., Capparelli R. (2011). Peptides from Royal Jelly: Studies on the antimicrobial activity of jelleins, jelleins analogs and synergy with temporins. J. Pept. Sci..

[26] Fujiwara S., Imai J., Yaeshima T., Kawashima T., Kobayashi K. (1990). A potent antibacterial protein in Royal Jelly: Purification and determination of the primary structure of royalisin. J. Biol. Chem..

[27] Álvarez-Suarez J.M., Gasparrini M., Forbes-Hernández T.Y., Mazzoni L., Giampieri F. (2014). The composition and biological activity of honey: A focus on Manuka honey. Foods.

[28] Hixon K.R., Bogner S.J., Ronning-Arnesen G., Janowiak B.E., Sell S.A. (2019). Investigating Manuka honey antibacterial properties when incorporated into cryogel, hydrogel, and electrospun tissue engineering scaffolds. Gels.

[29] Wallace A.J., Eady S.L., Miles M., Martin H., McLachlan A., Rodier M., Willis J., Scott R.S., Sutherland J. (2010). Demonstrating the safety of Manuka honey UMF^®^ 20+in a human clinical trial with healthy individuals. Br. J. Nutr..

[30] Hixon K.R., Lu T., Carletta M.N., McBride-Gagyi S.H., Janowiak B.E., Sell S.A. (2018). A preliminary in vitro evaluation of the bioactive potential of cryogel scaffolds incorporated with Manuka honey for the treatment of chronic bone infections. J. Biomed. Mater. Res. B Appl. Biomater..

[31] Hixon K.R., Lu T., McBride-Gagyi S.H., Janowiak B.E., Sell S.A. (2017). A comparison of tissue engineering scaffolds incorporated with Manuka honey of varying UMF. BioMed Res. Int..

[32] Candiracci M., Piatti E., Dominguez-Barragán M., García-Antrás D., Rodríguez-Morgado B., Ruano D., Gutiérrez J.F., Parrado J., Castaño A. (2012). Anti-inflammatory activity of a honey flavonoid extract on lipopolysaccharide-activated N13 microglial cells. J. Agric. Food Chem..

[33] Mesaik M.A., Dastagir N., Uddin N., Rehman K., Azim M.K. (2014). Characterization of immunomodulatory activities of honey glycoproteins and glycopeptides. J. Agric. Food Chem..

[34] Garraud O., Hozzein W.N., Badr G. (2017). Wound healing: Time to look for intelligent, ‘natural’ immunological approaches?. BMC Immunol..

[35] Okamoto I., Taniguchi Y., Kunikata T., Kohno K., Iwaki K., Ikeda M., Kurimoto M. (2003). Major Royal Jelly protein 3 modulates immune responses in vitro and in vivo. Life Sci..

[36] Majtan J., Kumar P., Majtan T., Walls A.F., Klaudiny J. (2009). Effect of honey and its major Royal Jelly protein 1 on cytokine and MMP-9 mRNA transcripts in human keratinocytes. Exp. Dermatol..

[37] Sugiyama T., Takahashi K., Tokoro S., Gotou T., Neri P., Mori H. (2011). Inhibitory effect of 10-hydroxy-trans-2-decenoic acid on LPS-induced IL-6 production via reducing IκB-ζ expression. Innate Immun..

[38] Biswas S.K. (2016). Does the interdependence between oxidative stress and inflammation explain the antioxidant paradox?. Oxidative Med. Cell. Longev..

[39] Hussain T., Tan B., Yin Y., Blachier F., Tossou M.C.B., Rahu N. (2016). Oxidative stress and inflammation: What polyphenols can do for us?. Oxidative Med. Cell. Longev..

[40] Kurek-Górecka A., Rzepecka-Stojko A., Górecki M., Stojko J., Sosada M., Świerczek-Zięba G. (2013). Structure and antioxidant activity of polyphenols derived from propolis. Molecules.

[41] Ilk S., Tan G., Emül E., Sağlam N. (2020). Investigation the potential use of silver nanoparticles synthesized by propolis extract as N-acyl-homoserine lactone-mediated quorum sensing systems inhibitor. Turk. J. Med. Sci..

[42] Al-Hatamleh M.A.I., Hatmal M.M., Sattar K., Ahmad S., Mustafa M.Z., Bittencourt M.D.C., Mohamud R. (2020). Antiviral and immunomodulatory effects of phytochemicals from honey against COVID-19: Potential mechanisms of action and future directions. Molecules.

[43] Vilchez A., Acevedo F., Cea M., Seeger M., Navia R. (2020). Applications of electrospun nanofibers with antioxidant properties: A review. Nanomaterials.

[44] Farokhi M., Mottaghitalab F., Reis R.L., Ramakrishna S., Kundu S.C. (2020). Functionalized silk fibroin nanofibers as drug carriers: Advantages and challenges. J. Control. Release.

[45] Melke J.J., Midha S., Ghosh S., Ito K., Hofmann S. (2016). Silk fibroin as biomaterial for bone tissue engineering. Acta Biomater..

[46] Cheng G., Davoudi Z., Xing X., Yu X., Cheng X., Li Z., Deng H., Wang Q. (2018). Advanced silk fibroin biomaterials for cartilage regeneration. ACS Biomater. Sci. Eng..

[47] Marrazzo P., Maccari S., Taddei A., Bevan L., Telford J., Soriani M., Pezzicoli A. (2016). 3D reconstruction of the human airway mucosa in vitro as an experimental model to study NTHi infections. PLoS ONE.

[48] Khan M.Q., Lee H., Khatri Z., Kharaghani D., Khatri M., Ishikawa T., Im S.-S., Kim I.S. (2017). Fabrication and characterization of nanofibers of honey/poly(1,4-cyclohexane dimethylene isosorbide trephthalate) by electrospinning. Mater. Sci. Eng. C.

[49] Lahooti B., Khorram M., Karimi G., Mohammadi A., Emami A. (2016). Modeling and optimization of antibacterial activity of the chitosan-based hydrogel films using central composite design. J. Biomed. Mater. Res. Part A.

[50] Mukhopadhyay A., Rajput M., Barui A., Chatterjee S.S., Pal N.K., Chatterjee J., Mukherjee R. (2020). Dual cross-linked honey coupled 3D antimicrobial alginate hydrogels for cutaneous wound healing. Mater. Sci. Eng. C.

[51] Wang T., Zhu X.-K., Xue X.-T., Wu D.-Y. (2012). Hydrogel sheets of chitosan, honey and gelatin as burn wound dressings. Carbohydr. Polym..

[52] El-Kased R.F., Amer R.I., Attia D., Elmazar M.M. (2017). Honey-based hydrogel: In vitro and comparative in vivo evaluation for burn wound healing. Sci. Rep..

[53] Park J.-S., An S.-J., Jeong S.-I., Gwon H.-J., Lee J.Y., Nho Y.-C. (2017). Chestnut honey impregnated carboxymethyl cellulose hydrogel for diabetic ulcer healing. Polymers.

[54] Tavakoli J., Tang Y. (2017). Honey/PVA hybrid wound dressings with controlled release of antibiotics: Structural, physico-mechanical and in-vitro biomedical studies. Mater. Sci. Eng. C.

[55] Rajput M., Mandal M., Anura A., Mukhopadhyay A., Subramanian B., Paul R.R., Chatterjee J. (2020). Honey loaded silk fibroin 3D porous scaffold facilitates homeostatic full-thickness wound healing. Materialia.

[56] Datta S., Sarkar R., Vyas V., Bhutoria S., Barui A., Chowdhury A.R., Datta P. (2018). Alginate-honey bioinks with improved cell responses for applications as bioprinted tissue engineered constructs. J. Mater. Res..

[57] Schuhladen K., Raghu S.N.V., Liverani L., Nescakova Z., Boccaccini A.R. (2021). Production of a novel poly(ɛ-caprolactone)-methylcellulose electrospun wound dressing by incorporating bioactive glass and Manuka honey. J. Biomed. Mater. Res. B Appl. Biomater..

[58] Kadakia P.U., Kalaf E.A.G., Dunn A.J., Shornick L.L., Sell S.A. (2018). Comparison of silk fibroin electrospun scaffolds with poloxamer and honey additives for burn wound applications. J. Bioact. Compat. Polym..

[59] Minden-Birkenmaier B., Neuhalfen R.M., Janowiak B.E., Sell S.A. (2015). Preliminary investigation and characterization of electrospun polycaprolactone and Manuka honey scaffolds for dermal repair. J. Eng. Fibers Fabr..

[60] Giusto G., Beretta G., Vercelli C., Valle E., Iussich S., Borghi R., Odetti P., Monacelli F., Tramuta C., Grego E. (2018). Pectin-honey hydrogel: Characterization, antimicrobial activity and biocompatibility. Biomed. Mater. Eng..

[61] Giusto G., Vercelli C., Comino F., Caramello V., Tursi M., Gandini M. (2017). A new, easy-to-make pectin-honey hydrogel enhances wound healing in rats. BMC Complement. Altern. Med..

[62] Bonifacio M., Cochis A., Cometa S., Scalzone A., Gentile P., Procino G., Milano S., Scalia A.C., Rimondini L., De Giglio E. (2020). Advances in cartilage repair: The influence of inorganic clays to improve mechanical and healing properties of antibacterial Gellan gum-Manuka honey hydrogels. Mater. Sci. Eng. C.

[63] Bonifacio M.A., Cochis A., Cometa S., Gentile P., Scalzone A., Scalia A.C., Rimondini L., De Giglio E. (2020). From the sea to the bee: Gellan gum-honey-diatom composite to deliver resveratrol for cartilage regeneration under oxidative stress conditions. Carbohydr. Polym..

[64] Schuhladen K., Mukoo P., Liverani L., Neščáková Z., Boccaccini A.R. (2020). Manuka honey and bioactive glass impart methylcellulose foams antibacterial effects for wound healing applications. Biomed. Mater..

[65] Kim J.I., Pant H.R., Sim H.-J., Lee K.M., Kim C.S. (2014). Electrospun propolis/polyurethane composite nanofibers for biomedical applications. Mater. Sci. Eng. C.

[66] Zhang T., Qu Y., Gunatillake P.A., Cass P., Locock K.E.S., Blackman L.D. (2020). Honey-inspired antimicrobial hydrogels resist bacterial colonization through twin synergistic mechanisms. Sci. Rep..

[67] Sharaf S., El-Naggar M.E. (2019). Wound dressing properties of cationized cotton fabric treated with carrageenan/cyclodextrin hydrogel loaded with honey bee propolis extract. Int. J. Biol. Macromol..

[68] González-Masís J., Cubero-Sesin J.M., Ureña Y.R.C., González-Camacho S., Mora-Ugalde N., Vega-Baudrit J.R., Loaiza R., Vega-Baudrit J.R., Gonzalez-Paz R.J. (2020). Increased fibroblast metabolic activity of collagen scaffolds via the addition of propolis nanoparticles. Materials.

[69] Simu M.-R., Pall E., Radu T., Miclăuș M.O., Culic B., Mesaros A.-S., Muntean A., Filip G.A. (2018). Development of a novel biomaterial with an important osteoinductive capacity for hard tissue engineering. Tissue Cell.

[70] Khodabakhshi D., Eskandarinia A., Kefayat A., Rafienia M., Navid S., Karbasi S., Moshtaghian J. (2019). In vitro and in vivo performance of a propolis-coated polyurethane wound dressing with high porosity and antibacterial efficacy. Colloids Surf. B Biointerfaces.

[71] Ahi Z.B., Renkler N.Z., Seker M.G., Tuzlakoglu K. (2019). Biodegradable polymer films with a natural antibacterial extract as novel periodontal barrier membranes. Int. J. Biomater..

[72] Ramírez O.J., Alvarez S., Contreras-Kallens P., Barrera N.P., Aguayo S., Schuh C. (2020). Type I collagen hydrogels as a delivery matrix for Royal Jelly derived extracellular vesicles. Drug Deliv..

[73] Lutolf M.P. (2009). Spotlight on hydrogels. Nat. Mater..

[74] Nayak A.K., Das B. (2018). Introduction to polymeric gels. Polymeric Gels.

[75] Bonifacio M., Cometa S., Cochis A., Gentile P., Ferreira A.M., Azzimonti B., Procino G., Ceci E., Rimondini L., De Giglio E. (2018). Antibacterial effectiveness meets improved mechanical properties: Manuka honey/gellan gum composite hydrogels for cartilage repair. Carbohydr. Polym..

[76] Bigham-Sadegh A., Torkestani H.S., Sharifi S., Shirian S. (2020). Effects of concurrent use of Royal Jelly with hydroxyapatite on bone healing in rabbit model: Radiological and histopathological evaluation. Heliyon.

[77] Petretto G.L., Cossu M., Alamanni M.C. (2014). Phenolic content, antioxidant and physico-chemical properties of Sardinian monofloral honeys. Int. J. Food Sci. Technol..

[78] Marrazzo P., O’Leary C. (2020). Repositioning natural antioxidants for therapeutic applications in tissue engineering. Bioengineering.

